# The application of large language models in pediatrics and medical research—Revolution or risk?

**DOI:** 10.1002/pdi3.39

**Published:** 2023-11-03

**Authors:** Janne Estill

**Affiliations:** ^1^ Institute of Global Health University of Geneva Geneva Switzerland; ^2^ School of Basic Medical Sciences Lanzhou University Lanzhou China

**Keywords:** academic writing, artificial intelligence, large language models, reporting guidance

## 1 INTRODUCTION

The emergence of artificial intelligence (AI) is certainly one of the most influential innovations of the present era. During the past years, applications of AI have transformed from experiments of a small group of experts into a part of everyday life. Large language models (LLMs), AI based tools that use natural language processing to generate human‐like dialogs, have, however, expanded the publicity and utilization of AI into a much broader audience than ever before, including researchers in different fields unrelated to computer science, other professionals needing to summarize information and produce written text, and also the general public who want to have easy answers to questions of everyday life. The release of ChatGPT in November 2022^1^ received particular publicity, but other LLMs have also become available on the market. The way LLMs act as if they were thinking like humans is fascinating and makes them easy to use regardless of the educational, cultural, and language background.

## 2 LARGE LANGUAGE MODELS AS A TOOL FOR WRITING ARTICLES

One of the most common applications of LLMs in research is its use for text generation and formatting. In the first issue of Pediatric Discovery, Leung and colleagues demonstrated the limitations of using ChatGPT to construct a narrative review on the immunogenicity of COVID‐19 vaccines, including an appendix describing the entire interaction with ChatGPT.^2^ The experience was controversial, showing that although there may be benefits for text formatting, ChatGPT was unable to produce, even with the assistance of the human authors, a solid, well‐structured article. The text produced by the tool was well formatted, but, nevertheless, contained, for example, irrelevant information despite the explicit instructions given by the authors, and the PubMed search queries were far from optimal, resulting in only very limited number of articles. Some of these problems may have been caused by technical limitations such as the restricted amount of input allowed and the inability to search latest content online, which can be expected to be overcome in the near future when more powerful versions and tools become available. However, this experience shows both a clear demonstration of the limitations as well as provides a good example on how to document and present the role of LLM tools in the formulation of the article.

## 3 GUIDELINES FOR THE USE AND REPORTING OF LARGE LANGUAGE MODELS ARE URGENTLY NEEDED

The widespread use of ChatGPT and other LLMs calls for strict regulation of their use. Recently, Kim and colleagues reviewed literature on the use of ChatGPT and proposed five statements for good practice, covering different aspects of transparency and scientific approach.^3^ Essentially, most of these statements reflect the fact that LLMs are only tools—LLMs should not be considered as authors, they should not be allowed to generate text without strict human verification, and their use should be clearly and transparently described. The fact that LLMs are easy to use without any specific training also needs caution—as suggested by Kim et al., researchers utilizing LLMs should also have at least a basic understanding of how the tool works. These statements are a good starting point to ensuring standardized procedures in the use of LLMs; however, it is also equally important that the entire process is transparently reported. Existing reporting checklists such as CONSORT, STROBE, PRISMA, and RIGHT^4^ were developed way before LLMs became in widespread use and thus do not yet cover these aspects. The WHO Collaborating Centre for Guideline Implementation and Knowledge Translation has suggested the development of a dedicated checklist for LLM utilization in research.^5^ The group has proposed a list of six initial items that should be clearly reported by any research utilizing LLMs in the production of the article, covering topics such as the details of the tool and its exact use, verification, and influence on the content. Although it will undoubtedly take some time before such a checklist is ready, it is already now the time for journal editors and reviewers to pay attention to the regulation of LLM use in research. Requirements to declare the use and exact role of any LLM tools in producing the article could be worth considering.

## 4 CAN LARGE LANGUAGE MODELS HELP PATIENTS AND CAREGIVERS TO SEEK MEDICAL ADVICE?

The use of LLMs is mostly discussed in the context of writing and presenting research, but it has also many other potential applications which may, if used inappropriately, cause serious risks. The use of LLMs by the general public can be seen as the successor for “Dr. Google.” However, the transition from basic internet queries to chats with LLMs will also lead to a substantial reduction in transparency. Whereas it is still relatively straightforward to recognize reliable web pages and online sources (e.g., websites of government health authorities, hospitals, and professional medical societies), this will not be the case with LLMs where the original source is usually unknown—in the worst case, the entire answer could be the result of “hallucination” by the LLM tool. Pediatrics is an especially sensitive field—small children are not able to clearly express the nature of their symptoms, and the consultation of a medical professional should always be the first action when there is any doubt of the condition. At the same time, we need to recognize the fact that LLMs, thanks to their user‐friendliness and ability to speak “the same language” with the patients and caregivers regardless of their educational background, have a great potential. However, general‐purpose tools like ChatGPT may not be the best option; instead, what is needed are tools dedicated for medical guidance, based on a solid scientific background that can be validated to be able to provide reliable answers to the users making queries related to their health status. An interesting opportunity could also be the use of LLM‐like tools for direct communication with children.

## 5 TRANSPARENCY AND SCIENTIFIC APPROACH NEED TO BE GUARANTEED AT ALL STAGES

In conclusion, we must bear in mind that AI is a much broader concept than ChatGPT or LLMs. AI has already been present for a long time in many fields of research. Machine learning methods are used to, for example, accelerate systematic reviews, deal with data sources that are too large to be analyzed using traditional methods, or to guide imaging in radiological processes. We need to, however, be critical when applying any AI methods. We must keep in mind—despite all the exciting opportunities that it offers—that AI is essentially a tool, not the purpose, nor the replacement for a human researcher or medical professional. The same principles that guide the use of any methods are also applicable for AI tools: the use needs to be transparent, clearly reported, and reproducible. The entire research community should work together to urgently build a regulatory framework that can support the scientific and efficient utilization of LLMs in medical research and decision‐making.

## AUTHOR CONTRIBUTIONS

**Janne Estill**: Conceptualization; literature search; writing the manuscript.

## CONFLICT OF INTEREST STATEMENT

The author serves as Deputy Editor‐in‐Chief of Pediatric Discovery.

## ETHICS STATEMENT

Not applicable.

## Data Availability

Data sharing not applicable to this article as no datasets were generated or analyzed during the current study.
